# Social and private activity after retirement—substitutes or complements

**DOI:** 10.1186/s12877-022-03464-y

**Published:** 2022-10-05

**Authors:** Małgorzata Kalbarczyk, Beata Łopaciuk-Gonczaryk

**Affiliations:** Faculty of Economic Sciences, University of Warsaw, Długa 44/50, 00-241 Warsaw, Poland

**Keywords:** Active aging, Social engagement, Social networks, Generalized Structural Equation Model

## Abstract

**Background:**

Commonly observed low activity of older adults harms their well-being. We perceive the retirement as a new opening that could be utilized to fulfill previously neglected needs and involve in new activities. They can be a remedy for losing the sense of life while changing the social role and getting older. This study explores trends in activity over retirement. In particular, it verifies if different post-retirement activities: 1) formal social engagement; 2) informal help given outside the household; 3) solitary leisure or self-development activities, and 4) sociable leisure or self-development activities are mutually exclusive or supportive of each other.

**Methods:**

We use the data from 4 and 6th wave of Survey of Health, Ageing and Retirement in Europe, taking into consideration 2757 respondents who participated in both waves. We analyze trends in activity over retirement and verify the hypotheses regarding the relationships between engaging into different types of activities and between the activity and development of personal social networks. We conduct the descriptive analysis and utilize the Generalized Structural Equation Model.

**Results:**

Most people do not change their involvement in different activities after retirement. Among those who change, the average trend is upward. We find support that different types of activities undertaken after retirement do not crowd out each other. On the contrary, being active in one sphere goes hand in hand with activity in another. We give evidence for presence of social network mechanisms that enhance such understood complementarity. Maintaining social relationships correlates with engagement in socially-oriented activities and privately-oriented sociable pastimes. Being active, with exception of solitary forms of recreation, creates an opportunity to foster relations with other people and, thanks to their encouragement, to engage in new activities.

**Conclusions:**

The small change in activity after retirement signifies the need to encourage non-work activity during earlier stages of life. As different types of post-retirement activities are complementary, the most active group of older adults would be the most open for social engagement and volunteering, however the least active group needs the biggest support to involve in any activities.

## Introduction

Retirement can be a life-changing moment, due to a need of adjustment to a new social role, associated with gaining a new perspective and increasing the amount of spare time, which can be used to fulfill needs and aspirations that were neglected previously. This can be connected with more social engagement and increased involvement in helping other people, or undertaking activities aimed at self-development and entertainment.

The main aim of this study is to verify if different types of activities that people engage in after retirement: 1) formal social engagement in voluntary and charity work, and political or community related organizations (formal social activity); 2) informal help given outside the household (informal social activity); 3) solitary leisure or self-development activities (private solitary activity) and 4) sociable leisure or self-development activities (private sociable activity) are either mutually exclusive (substitutes) or supportive (complements) of each other. Substitution would mean that different groups of older adults specialize in different types of activities following their personal preferences and, due to the time constraints, different activities crowd out each other. Complementarity would be associated with the situation, where engagement in one type of activity enhances undertaking another which enables fulfillment of different needs.

Additionally, we want to inquire about the role played by maintaining relationships within the structure of a personal social network. First of all, having different types of social connections is associated with engagement in different activities. We expect that family ties are more connected to informal social activity, while friends and acquaintances’ ties are more associated with formal social activity. This could lead to a tendency to choose different activities by older adults with a different composition of social networks. Secondly, through the development of relationships within social networks, different types of activities may become complementary. Being active, with exception of solitary forms of recreation, creates a chance to foster relations with other people and, thanks to their encouragement, to engage in new pastimes. This can lead to complementarity between informal social activity and private sociable activity, as well as formal social activity and private sociable activity, assuming that private sociable activity is associated both with relationships with family and friends.

Our study contributes to the existing literature in the following aspects. Firstly, we focus on the time of retirement as the moment of a possible life change, while most of the other studies generally look at older adults. We investigate factors which determine not sole participation in different activities, but the potential increase in activity after retirement. Secondly, most of the existing literature takes into consideration only one type of activity at a time (like volunteering or leisure activities) while we analyze many different activities at the same time and look at their mutual relationships. Thirdly, we pay special attention to the role of personal social networks and verify if they enforce substitution or complementarity between different older adults’ activities.

The problem we address regards low activity of older adults which harms their well-being. In EU-27 countries in 2015 only about 11% among 65 year or older got together on a daily basis with friends, 2.9% participated in formal and non-formal education and training and less than a half participated in cultural and sport events or in tourism [1]. Simultaneously less than 10% of people above 65 years provided informal homecare services to both someone in the same household and to someone in a different household. At the same time, there are evidences that greater leisure activity [2] or volunteering [3] increase the sense of purpose in life and for some people can be a compensation for withdrawing from professional career. We perceive the moment of transformation into retirement as connected with a potential motivation for engaging in new activity, which can pay off in later years. We want to explore the patterns of potential increase in different types of activities after retirement and its determinants. Enhancing such understanding should be useful and interesting in the context of designing policies supporting active aging.

## Literature review

### Role of retirement transition in activity changes

Transition into retirement can be analyzed based on the role theory [4], because it is associated with abandoning or weakening the professional career role and a need of adjustment to a different social role [5]. This may translate into strengthening the roles associated with family and social environment, as retirement creates an opportunity for larger involvements in family life and activities benefiting the community [6]. Furthermore, under the alternative conceptualization, the continuity theory [7], we may claim that successful adaptation into new life circumstances needs pre-retirement non-work activity (like developing hobbies) and is supported by previous social relations and group membership [8]. However, it is justified to perceive the transition into retirement as a life changing moment regarding the non-work activity, as there is a tendency to postpone leisure and social goals until retirement [9].

There are only a few studies that indicate a change in the activity of older adults at a specific moment of retirement, and not just activities of older adults above a certain age. Existing research focuses most of all on the relationship between retirement and a change in involvement in volunteering and informal care, and participation in organizations, and pays less attention to the privately-oriented activities. For example, Sabbath et al. [10] analyze changes in active participation in organizations after retirement and find out the important role played by gender, self-assessed health, and midlife socioeconomic status.

Erlinghagen [11] shows on the German Socio-Economic Panel (SOEP) database that retirement does not have an effect on the participation in voluntary or informal help. Previous voluntary or help experience is more important. Similar results regarding volunteering and informal help after retirement were obtained on the data of Americans’ Changing Lives survey [12]. However, besides these evidences for continuity, retirement is also found to be associated with an increased chance for having a hobby [13]. Retirement goes hand in hand with an increase in recreational and household activity, as well as TV watching [14]. It also can have an effect on the increased engagement in volunteering, in order to compensate for the withdrawal from the professional job role [15]. Another study confirms its positive impact not only on formal volunteering and organizational participation, but also informal help towards family and friends [16].

### Substitution and complementarity between the activities

An analysis of older adults’ activity cannot avoid discussing the possibility of involvement in a variety of activities simultaneously. These activities can impede or stimulate each other.

Time constraints can impede the choice of different activities by older people. Choi et al. [17] show on a Health and Retirement Study (HRS) database that wives who care for their husbands are less likely to provide formal or informal help to others. Similarly, Kamakura [18] or McNamara and Gonzales [19] indicate that employment or providing care to others reduces time left to participate in a voluntary service. Pettigrew et al. [20] state that for older Australians, temporal factors are important in assessing their ability to participate in volunteering. Additionally, Morrow-Howell et al. [21] show that among American Experience Corp Program participants, volunteering does not happen in isolation from other activities and among highly active people, more time devoted to volunteering results in less time left for other activities.

Except for time and other resources’ constraints, also individual preferences can cause specialization of older adults in the choice of the type of activity they want to engage into, selecting between privately- and socially-oriented activities and their different sorts. This is in accordance with the idea of some people being more focused on self-interest and the other on the welfare of others [22–24]. Even if obtaining private benefits can be a reason not only to get involved in the leisure and self-development, but also in socially-oriented activities like volunteering [25], still the last are additionally associated with the motivation regarding the care for others and therefore more suitable for people with altruistic preferences.

However, there is also much evidence supporting complementarity of different activities undertaken, which may result from the individual personal traits indicating that there are active and inactive persons. Taking part in an activity provides a chance to meet people, which in turn creates opportunities for participation in other activities [26]. Both Hank, and Stuck [27] and Kohli et al. [28] show on Survey of Health, Ageing and Retirement in Europe (SHARE) data that formal and informal care go hand in hand. Similarly, for England, van der Horst et al. [29] show on the English Longitudinal Study of Ageing (ELSA) panel data that part-time paid work is complementary with volunteering. Nazroo [30], also for England, concludes that participation in volunteering and work itself is complementary, while the time devoted to both activities is substitutive. However, apart from this research there is a lack of discussion regarding a possibility of complementarity or substitution between a variety of activities which can be performed by older adults.

Different kinds of activities can follow different motivations, corresponding to different psychological traits. In the context of the proposed division between socially and privately-oriented activities, it is useful to refer to the notions of communion and agency, the first associated with connection to a group or society, and the second associated with striving for individual achievements [31]. High communion can coexist with high agency, leading to fulfillment of different needs which constitutes the core of successful aging conceptualization [32] and supporting the prediction of complementarity between different types of activities. Communion will be associated with activities involving care for others, while agency can be demonstrated in individual practicing tai chi, both in the case of the same, “thriving” old woman [33].

Building on the presented literature background, we predict complementarity between different forms of older adults’ activity, in accordance with the concept of successful aging connected with fulfillment of different needs. Finding a balance between incentives connected both with agency and communion leads to psychological well-being as it enables both obtaining satisfaction with own achievements and providing for the next generation [34]. This results in the following expectation:

- Hypothesis 1: Increase in engagement into one type of activity is associated with the increase in participation in the other type of activity.

### Role of social networks

Post-retirement activity can be expected to highly correspond to personal social networks and their change. Personal social networks of older adults correlate with socially oriented activity such as participation in voluntary associations [28]. This relationship can be two-sided: for example those with larger social networks tend to have a higher civic engagement and this engagement, in turn, leads to establishing larger social networks [35]. It is specially expected to be connected with friends and acquaintances ties, and not family ties. Non-kin relationships are associated with a broader radius of trust and therefore can increase openness for needs of strangers and involvement in formal civic organizations [36, 37]. At the same time, informal help is especially expected within the family. It is often intergenerational, between children and parents [38–40].

Older adults’ family ties and friends and acquaintances’ ties are found to be negatively correlated [28]. Similarly, loss of a family member can be followed by an increase in community involvement, which serves as a way of compensation and adaptation [35]. This is especially evident in case of increased involvement in volunteering after spousal bereavement [41].

Social networks influence not only socially-oriented activity, but also can foster privately-oriented activity, for example in the form of engagement in art and craft [42]. It is plausible both in case of family and friends and acquaintances’ ties.

The role of social networks for older adults is culturally bounded and may differ between countries [43]. For example, in Central and Eastern European countries social networks are connected mainly with informal help but not participation in voluntary organizations, and in Scandinavian countries social care is mostly provided by government assistance and not the relatives.

Developing social relationships contributes to participation in activities and can potentially enhance the complementarity between different types of post-retirement activity. Joining one kind of activity translates into creating relations with people who encourage engagement in other activities. Therefore retirement can be an opportunity to increase both social connectedness and community involvement. It translates into the following expectations:

- Hypothesis 2.1: Increase in family network size is associated with the increase in informal help (which in turn correlates with the increase in involvement in other types of activities, as assumed in Hypothesis 1).
- Hypothesis 2.2: Increase in family network size is associated with the increase in privately-oriented sociable activities (which in turn correlates with the increase in involvement in other types of activities, as assumed in Hypothesis 1).
- Hypothesis 2.3: Increase in friends and acquaintances’ network size is associated with the increase in formal social engagement in organizations (which in turn correlates with the increase in involvement in other types of activities, as assumed in Hypothesis 1).
- Hypothesis 2.4: Increase in friends and acquaintances’ network size is associated with the increase in privately-oriented sociable activities (which in turn correlates with the increase in involvement in other types of activities, as assumed in Hypothesis 1).
- Hypothesis 2.5: Increase in informal help is associated with the increase in family network (due to two-way dependency), which in turn correlates with the increase in privately-oriented sociable activities (possible way of spending time with family).
- Hypothesis 2.6: Increase in formal social engagement in organizations is associated with the increase in friends and acquaintances’ network (due to two-way dependency), which in turn correlates with the increase in privately-oriented sociable activities (possible way of spending time with friends and acquaintances).
- Hypothesis 2.7: Increase in privately-oriented sociable activities is associated with the increase in family network (opportunity to enhance relations), which in turn correlates with the increase in informal help.
- Hypothesis 2.8: Increase in privately-oriented sociable activities is associated with the increase in friends and acquaintances’ network (opportunity to foster relations), which in turn correlates with the increase in formal social engagement in organizations.

## Methods

### The database and the sample extracted

The data under analysis has been obtained from the publicly available database SHARE: Survey of Health, Ageing and Retirement in Europe (available on www.share-project.org). The SHARE database is a biennial panel study conducted on a representative sample of people aged ≥ 50 years across 20 European countries [44]. The study brings together many disciplines, including demography, economics, epidemiology, and psychology. The data contains information regarding respondents’ professional status, social activities, satisfaction, functional capacity as declared by the respondents, and support received and given. In this study, we used release 6.1.1 of wave 4 and wave 6 of the survey, which was conducted in 2011 and 2015, respectively.

The sample covers all respondents who, according to the data from the 6^th^ wave (from 2015), declared that they went into retirement in 2012, 2013, and 2014, which is after the 4^th^ wave (from 2011). The initial focus on the change between the 4^th^ and 6^th^ waves is caused by the fact that the social network questionnaire was performed only during these two waves. Variables on social engagement and other activities performed during the last year, life satisfaction, and life conditions are taken from both the 4^th^ and 6^th^ wave. Only respondents who took part in both the 4^th^ and 6^th^ wave are included. People declared as residents of nursing homes are excluded. The constructed database covers a total of 2757 respondents from 14 countries. There are some missing values, and their amounts are different for different variables. Adequate sample sizes are indicated while presenting the results of the analyses.

### Description of variables

We distinguish between four types of activity: formal and informal socially-oriented activity, and solitary and sociable privately-oriented activity. All activities are related to the past twelve months. Table 1 presents a description of variables.

**Table 1:** Description of variables

Formal social activity	includes: -volunteering, indicating respondents who did voluntary or charity work -civic organizations, indicating respondents who have taken part in a political or community-related organization
Informal social activity	respondents who gave help (personal care or practical household help) to people outside their household
Private solitary activity	includes: -books’, magazine or newspaper reading -word and number games like crossword puzzles or Sudoku
Private sociable activity	includes: -sport or social club, indicating respondents who went to a sport, social or other kind of club -educational course, indicating respondents who attended an educational or training course -cards or chess indicating respondents who played cards or games such as chess
Increase in informal social activity	1- a respondent, after retirement, started to be engaged in informal help given outside the household 0- a respondent didn’t engage in this activity
Increase in formal social activity	1- a respondent, after retirement, started to be engaged in one or both of the activities from this type 0- a person joined one activity but resigned from the second, or didn’t join any of those activities
Increase in private solitary activity	1- a person, after retirement, started to be engaged in one or both of the activities from this type 0- a person joined one activity but resigned from the second, or didn’t join any of those activities
Increase in private sociable activity	1- a person, after retirement, started to be engaged in one or more of the activities from this type 0- a person engaged in some activities and resigned from some others (the number of activities up-taken versus the number of activities abandoned is considered to decide if there was an increase or not) or didn’t join any of those activities
Family^a^	number of family members with whom respondents most often discuss important things
Friends & acquaintances	number of non-kin with whom respondents most often discuss important things
Age	difference between year of interview and year of birth
Females	0- men 1- women
Years of education	number of years of education
Life satisfaction	0- completely dissatisfied to 10-completely satisfied
Living in the country	0- living in a town or city 1- living in the country
Household size	number of household members
Income	logaritmized value of income per household member
Trusting people	0- can’t be too careful to 10-most people can be trusted
Praying	0- respondent who never prays 1- respondent who prays at least sometimes
Received help	0- respondent’s household didn’t receive help from another household 1- respondent’s household received informal help (personal care or practical household help) from another household
Limitations in ADL	count of limitations in Activities of Daily Living — basic self-care tasks
Limitations in IADL	count of limitations in Instrumental Activities of Daily Living — e.g. shopping or preparing meals
Limitations	count of limitations in Activities of Daily Living and Instrumental Activities of Daily Living
Active countries	1- countries where mean activity after retirement (within the analyzed sample) is higher than mean in the whole sample (Austria, Belgium, Denmark, France, Germany, Sweden, and Switzerland) 0- other countries (Czech Republic, Estonia, Italy, Poland, Portugal, Slovenia and Spain)

Source: Own Study

^a^family and friends & acquaintance variables sum up to 7 nominations

All variables regarding the increase in activity after retirement are constructed by comparison of the data from 2011 and 2015 (and knowing that, due to our sample design, the transition into retirement took place between these two points in time).

Apart from variables of our direct interest, connected with participation in different activities and maintaining social relationships, we have decided to include in the statistical modeling several control variables: age, females, years of education, life satisfaction, living in the country, household size, income per household member (logarithmized in GSEM model), trusting people and praying. This choice was based on the literature regarding determinants of broadly understood active aging [10, 19, 26, 27, 45–51].

### Methodological approach

**Fig. 1:**
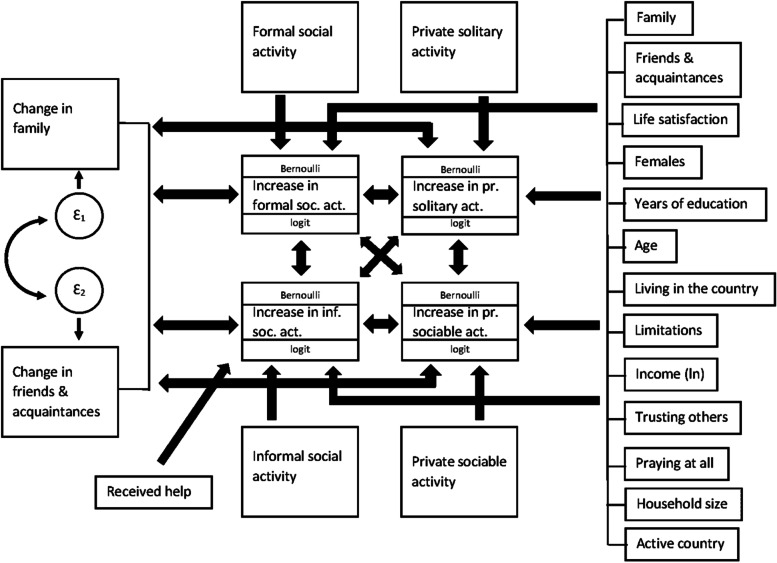
GSEM path diagram. Source: Own Study

Our methodological strategy is as follows. As a start, we look at the differences between the activities of people before and after retirement (that is in 2011 and 2015) and the analogical change in other variables under analysis. Then we estimate a statistical model in order to verify multiple relationships between increases in different types of activities after retirement, taking into consideration the role of social networks and controlling other possible determinants of upward changes in older adults’ activity. We utilize a GSEM approach, which can be conceptualized as the generalization of structural equation modeling (SEM), allowing the use of discrete variables and the estimation of logistic equations in modeling [52]. The advantage of this approach is the possibility to simultaneously estimate multiple equations. This allows us to explore associations between multiple variables in the same model. The GSEM model, presented as a path diagram on Fig. 1, has been estimated using the SHARE database. Included equations are logistic regressions (for increases in different types of activities) and linear regressions (for change in family and friends & acquaintances social network) estimated simultaneously by using a maximum-likelihood estimator, with standard errors corrected by clustering observations by respondents’ country of residence. The model assumes that an increase in one type of activity can affect involvement in the other. We are interested in the increase in activity after retirement, because we perceive it as a possible form of adaptation and compensation regarding negative sides of retirement [48].

“Increase” and “change” in variables refer to the change between and after retirement (which is between 2011 and 2015)^1^. All other variables refer to the pre-retirement state, which is in 2011.

We use the same set of determinants for the dependent variables concerning increase in four types of activities, except for support received from other households included only in the equation for informal social activity. Regressions explaining change in family and friends/acquaintances networks enable us to test the expected two-side relationship between increase in activity and increase in social networks. By adding the covariance between the residuals of these regressions, we predict negative correlation between family and friends/acquaintances networks, based on literature [28, 53].

All estimations were performed in STATA software.

## Results

### Descriptive analysis

As it can be concluded from Table 2, after retirement, respondents are more active in voluntary and charity work, helping others, reading books and magazines, doing word and number games, clubs, and playing cards or chess. In contrast, they spend less time in educational and training courses, which may be more connected with performing work. An increase in participation in civic organizations is not significant and this activity is rather rare among participants. Their social networks get enlarged, but this is particularly true about their relationship with family members. This may be explained by the fact that the network of friends and acquaintances may be grounded in work relations [54]. An increase in social networks among older adults, with an increasing share of family ties, was also reported by [55]. Additionally, over retirement, respondents are generally more satisfied with life. Furthermore, different aspects of life conditions deteriorate over time after going into retirement, namely: health and average income per person. There is also a tendency to move to the countryside, have a smaller household size and receive more help as a household.

**Table 2:** Change in activities and respondents’ characteristics after going into retirement, 14 countries, paired samples

	Mean or percent of positive answers:			
	before retirement	after retirement	(obs.)	
Volunteering (binary)	16.94	20.01	(2697)	***
Civic organizations (binary)	6.9	7.16	(2697)	
Gave help (binary)	31.76	36.32	(1817)	***
Books or magazines (binary)	78.46	81.72	(2697)	***
Word or number games (binary)	43.05	49.94	(2697)	***
Educational course (binary)	17.54	11.75	(2697)	***
Sport or social club (binary)	29.48	31.89	(2697)	***
Cards or chess (binary)	30.66	34.82	(2697)	***
Size of social network (0–7, mean)	2.57	2.77	(2721)	***
Family (0–7, mean)	1.86	2.10	(2721)	***
Friends and acquaintances (0–7, mean)	0.50	0.53	(2721)	*
Life satisfaction (0–10, mean)	7.72	7.82	(2681)	***
Living in the country (binary)	37.30	38.70	(2646)	**
Limitations in ADL (0–6, mean)	0.11	0.13	(2760)	***
Limitations in IADL (0–9, mean)	0.11	0.21	(2760)	***
Received help (binary)	0.13	0.19	(1817)	***
Income (mean, 0.02–128,745.30)	2340.72	1990.79	(1987)	**
Household size (0-, mean)	2.3	2.2	(2766)	***

Source: Authors’ own analysis based on SHARE wave 4 and 6, release 6.1.1

Significance levels: *** *p* < 0.01, ** *p* < 0.05, * *p* < 0.1, for binary variables: 1 = selected, 0 = not selected, don’t know = lack of data

In addition to the presented average changes, it has to be underlined that most of the respondents who were active before retirement remained active, and those who were inactive remained inactive. For formal social activities, 79% of people did not change their engagement. For other types of activities, the following shares of respondents retained the same activity after retirement: 63% in case of informal social activities, 61% for private solitary activities and 56% for private sociable activities.

### Results from the GSEM

The estimated GSEM identifies determinants of the growth of engagement in the four considered types of activity and the reciprocal relationship from the increase in activity towards the change in social networks (Table 3).

**Table 3:** Determinants of increase in different types of activity and change in social network — results from the GSEM

VARIABLES	Informal social activity	Formal social activity	Private solitary activity	Private sociable activity	Change in friends & acquaintances	Change in family
Increase in priv. solit. act	0.046	0.381**		0.139	0.103	0.052
	(0.289)	(0.175)		(0.192)	(0.073)	(0.164)
Increase in priv. soc. act	-0.217	0.162	0.471**		0.081	0.137*
	(0.290)	(0.178)	(0.178)		(0.059)	(0.078)
Increase in inform. soc. act		0.185	0.510	-0.017	0.224***	0.295***
		(0.315)	(0.424)	(0.259)	(0.080)	(0.067)
Increase in formal soc. act	0.444		0.427*	0.284	0.153*	0.050
	(0.330)		(0.247)	(0.187)	(0.082)	(0.117)
Family	0.260***	0.042	0.042	0.147		
	(0.080)	(0.084)	(0.090)	(0.101)		
Friends & acquaintances	0.046	0.189	-0.097	0.070		
	(0.214)	(0.119)	(0.167)	(0.128)		
Change in friends & acquaintances	0.355**	0.266**	-0.088	0.145		
	(0.180)	(0.129)	(0.169)	(0.168)		
Change in family	0.256***	0.092	-0.053	0.104*		
	(0.071)	(0.127)	(0.069)	(0.058)		
Age	0.022	0.014	0.024***	0.040***		
	(0.015)	(0.026)	(0.008)	(0.016)		
Years of education	0.071***	0.086***	0.035	0.067***		
	(0.019)	(0.028)	(0.037)	(0.018)		
Females	-0.015	-0.204	0.490***	0.082		
	(0.267)	(0.278)	(0.184)	(0.186)		
Living in the country	0.300	0.130	-0.062	0.062		
	(0.257)	(0.294)	(0.210)	(0.283)		
Household size	-0.199*	-0.041	0.037	0.002		
	(0.120)	(0.122)	(0.095)	(0.058)		
Life satisfaction	0.071	0.081	-0.069	0.048		
	(0.051)	(0.058)	(0.065)	(0.030)		
Trusting people	-0.050	0.041	0.008	-0.038		
	(0.068)	(0.044)	(0.053)	(0.036)		
Praying	-0.177	0.816**	-0.430	0.050		
	(0.326)	(0.327)	(0.335)	(0.128)		
Limitations	-0.193	-0.255**	0.038	-0.227*		
	(0.136)	(0.127)	(0.032)	(0.136)		
Income (ln)	0.045	0.081	-0.046	-0.031		
	(0.029)	(0.069)	(0.090)	(0.092)		
Active country	0.339	0.501**	0.577**	0.660***		
	(0.369)	(0.243)	(0.254)	(0.227)		
Received help	0.543**					
	(0.250)					
Constant	-46.46	-33.30	-46.68***	-85.85***	-0.12***	0.04
	(29.72)	(50.98)	(14.65)	(31.16)	(0.04)	(0.08)
Residuals covariance (regarding the change in social networks)					-0.19*** (0.03)	
Observations	1684	1684	1684	1684	1684	1684

Source: Authors’ own analysis based on SHARE wave 4 and 6, release 6.1.1

Robust standard errors in parentheses. Significance levels: *** *p* < 0.01, ** *p* < 0.05, * *p* < 0.1. Country reference group including Poland, Czech Republic, Portugal, Italy, Spain, Slovenia and Estonia. In all models, variables controlling level of adequate activity in first period are included

The results obtained from the GSEM show complementarity between formal social activity and private solitary activity (the increase in private solitary activity positively correlates with the increase in formal social activity), and between private solitary activity and private sociable activity (the increase in private sociable activity positively correlates with the increase in private solitary activity). This gives support to Hypothesis 1. 

The pre-retirement number of family members in the social network and its change through retirement correspond, as expected in Hypothesis 2.1, to engaging in post-retirement informal social activity. Additionally, also in accordance with our expectations, an increase in family network goes hand in hand with the increase in private sociable activities. This confirms Hypothesis 2.2.

Enlarging the number of friends and acquaintances in a social network through retirement correlates with higher involvement in both informal and formal social activity after retirement, which is in line with results obtained by other authors [47, 50] and partly according to our assumptions, regarding solely formal social activity (Hypothesis 2.3). Furthermore, we do not find support for our expectations formulated in Hypotheses 2.4, as the increase in friends and acquaintances network’s size and the increase in private sociable activities are not correlated.

The increase in different activities is correlated with changes in the number of members in family and friends & acquaintances social networks. As expected within Hypothesis 2.5 and 2.7, increasing private sociable and informal social activities is associated with growth in family network size. Increases in formal and informal social activities correspond to a positive change in friends & acquaintances, which is partly to our assumptions (formulated in Hypothesis 2.6), as we do not predict here the significance of informal social activities, which we relate to family networks. However we observe no effect of increase in private sociable activities on growth of friends & acquaintances network, against the assumptions in Hypothesis 2.8.

## Discussion

According to our analysis we can observe that both activities and social networks are changing as a result of retirement and the main trend is here upward. However these changes are not big and the general picture is that active people remain active and inactive people remain so after retirement. We observe complementarity in the increase in involvement in various types of activity. The analogous results, but for formal and informal social activities, were obtained by other authors [17, 27, 28, 30, 47].

Similar to our study, an analysis conducted by Dury et al. [59] for Belgium shows the complementarity of volunteering not only with other social activities, but also with private activities.

Table 4 presents the verification of the hypotheses: Hypothesis 1 — regarding the association between increases in different types of activities, and Hypotheses 2.1–2.8 — regarding the network mechanisms leading to the complementarity of the undertaken activities.

**Table 4:** Verification of hypotheses

Hypothesis 1	increase in private sociable activity ⇒ increase in private solitary activity increase in formal social activity ⇔ increase in private solitary activity	confirmed
	association between increases in other pairs of activity types	not confirmed
Hypothesis 2.1	increase in family network ⇒ increase in informal social activity ⇒ increase in other activity	not fully confirmed (increase in informal help does not foster increase in any other type of activity)
Hypothesis 2.2	increase in family network ⇒ increase in private sociable activity ⇒ increase in other activity (private solitary activity)	confirmed
Hypothesis 2.3	increase in friends and acquaintances network ⇒ increase in formal social activity ⇒ increase in other activity (private solitary activity)	confirmed
Hypothesis 2.4	increase in friends and acquaintances network ⇒ increase in private sociable activity ⇒ increase in other activity (private solitary activity)	not fully confirmed (increase in friends and acquaintances network does not foster increase in private sociable activity)
Hypothesis 2.5	increase in informal social activity ⇒ increase in family network ⇒ increase in private sociable activity	confirmed
Hypothesis 2.6	increase in formal social activity ⇒ increase in friends and acquaintances ⇒ increase in private sociable activity	not fully confirmed (increase in friends and acquaintances network does not foster increase in private sociable activity)
Hypothesis 2.7	increase in private sociable activity ⇒ increase in family network ⇒ increase in informal social activity	confirmed
Hypothesis 2.8	increase in private sociable activity ⇒ increase in friends and acquaintances network ⇒ increase in formal social activity	not fully confirmed (increase in private sociable activity does not foster increase in friends and acquaintances network)
Additional mechanism fostering complementarity, not predicted in the hypotheses	increase in formal social activity ⇒ increase in friends and acquaintances ⇒ increase in informal social activity	confirmed

Source: Own Study

Our findings are in accordance with the “successful aging” research framework [25, 32]. We present evidence that increase in one activity corresponds to the increase in another, however we identify this complementarity only between some types of activity as no direct correlation between increase in informal social activity and other types of activity is found.

We succeed in positively verifying at least some of the expected social network mechanisms fostering the complementarity. We confirm two mechanisms based on the role of social networks in increasing the activity in one domain and therefore corresponding to the increase of the activity in another domain (Hypothesis 2.2 and 2.3). We also give evidence supporting two network mechanisms leading to indirect complementarity between different types of activities (informal social activity and private sociable activity), through mediation of social network (Hypothesis 2.5 and 2.7).

Positive relation between family network and engagement in helping others gives evidence supporting our expectations that family ties can potentially increase informal social activity. We also have found positive correlation between gaining new friends and acquaintances and increased engagement in both informal and formal social activity, although we have here predicted the significance of the second case, connected with participation in civic organizations. This may demonstrate that informal help is not only expected in family, but also between friends. Taking into consideration that respondents nominated only up to 7 members of their social network in total (including kin and non-kin), it is probable that it consists of close friends rather than distant acquaintances. However there is also a possibility of informal help outside households not being limited to closest friends and kin, as an alternative to less popular formal help.

According to our results, older respondents are more likely to increase their solitary and sociable private activities. Cornwell et al. [35] show a positive relationship between age and volunteering, which has not been confirmed in our study. A higher probability of an increase in formal and informal social activity and private sociable activity after retirement is observed as education increases, which is in line with the results of other studies [26, 27, 45–47, 49, 50]. Females are more likely to increase private solitary activity. A lower activity of women in organizational activities after retirement is found by Sabbath et al. [10] in France. People who pray are more likely to increase their engagement in formal social activities after retirement, which is opposite to the findings presented by Crosnoe and Elder [48], indicating that there is no association between a successful, well-rounded aging lifestyle and that with religious involvement, but in line with results obtained by Butrica et al. [26]. In addition, for people with more ADL or IADL difficulties, the probability of an increase in formal social or private sociable activity is lower. It means that people with functional difficulties are less likely to be active outside the home and supports the results presented in the literature [10, 48]. People whose household has received help were more likely to help others after retirement, which is consistent with the concept of reciprocity and the findings regarding the interplay between giving and taking in social networks of older people [56]. Having a smaller household correlates with starting to help outside the household more often, which is probably associated with less involvement in the assistance required inside the household.

We find a significant effect of active country for growth of engagement in both private activities and formal social activity, which can be explained by two reasons. First, in countries with high average activity of older adults, we can expect social norms and customs, as well as peer-effects, that support participation. Second, in those countries older adults have more appropriate resources, both material and connected with better health thanks to better health care, and better access to potential activities with more options to choose from. International differences were also stated by Hank [57] who observes greater engagement in productive aging in countries with greater political and religious freedom and more provision of welfare service. Interestingly, a retiree’s financial situation has no impact on the probability of an increase in any activity after retirement. According to the literature, income should have influence on cultural and recreational activities [58].

Our study has several limitations due to the available dataset and the methodological approach taken. First, our sample was limited to people who participated both in the 4th and 6th wave of the SHARE study, which allowed us to analyze changes in activity after retirement, but can lead to selection bias. Additionally, we do not have data enabling to observe the time sequence of changes in activity. As we assess all changes at the same time, it is not possible to distinguish which one was the prior, and which was the subsequent.

Second, due to the small number of observations, analyses for individual countries were impossible, therefore we considered the groups of countries. Third, we were not able to analyze informal help provided within households. Further, the choice of activities considered within each of the proposed type of activities was restricted due to availability of data, and we omitted many potential activities, e.g. gardening or taking care of pets. We also did not control various time consuming activities, like housework. Last but not least, there is an increasing number of people who continue work after retirement which constrains their possibilities of undertaking other activities. Additionally, transition into retirement is not always a definite transfer, as we assumed for simplification, but can be a process following different phases, and the increased engagement in activities straight after retirement does not have to be long-term. Future studies could include the missed out activities, with a special consideration for the professional activity after retirement. This study focused on the activity right after retirement, it would be beneficial to examine the behavior of retired people over a longer time horizon. A promising avenue for future research is also a study of post-retirement activity within a framework of social networks’ dynamics, while considering different, both closer and more distant social circles.

## Conclusions

Our study shows that retirement is not a factor that can dramatically change peoples’ choice of the non-work activities they engage in. Furthermore, we have not observed any evidence that various types of activities are substitutes, which could be expected because of possible time constraints and individual preferences. On the contrary, we show that different post-retirement activities can be complementary, in accordance with the “successful aging” idea regarding involvement in different types of activities fulfilling different needs. With support of similar results from literature [59], we state that privately-oriented activity does not crowd out socially-oriented activity and vice versa. This may indicate that primary interest should be about retired people being active at all, and not specifically about their social engagement. The implications are that there are general barriers for older adults’ activity, and not conflicts between its particular types. It seems to be not only a problem of financial and time constraints (having to choose between possible activities) but also psychological and sociological issues (connected with willingness to engage).

Our study contributes to the present literature by the simultaneous consideration of a wide range of activities, testing the concept of substitution versus complementary and analyzing the effects of social networks’ type as possible mechanisms enhancing the post-retirement activity. The results support several possible policy recommendations. The small change in activity after retirement may suggest the need to encourage non-work activity during earlier stages of life. As different types of activities are complementary, it is important to consider older adults’ activity as a complex concept even if the primary interest could be in socially-oriented activities, as they benefit the whole community. The most active group of older adults would be the most open for social engagement and volunteering, however the least active group is in the most need of support to get active in any sphere of life.

## Data Availability

The data under analysis has been obtained from the publicly available database SHARE: Survey of Health, Ageing and Retirement in Europe, http://www.share-project.org/data-access/user-registration.html. This paper uses data from SHARE Waves 4 and 6 (DOIs: 10.6103/SHARE.w4.800, 10.6103/SHARE.w6.800). The SHARE data collection has been funded by the European Commission, DG RTD through FP5 (QLK6-CT-2001–00,360), FP6 (SHARE-I3: RII-CT-2006–062,193, COMPARE: CIT5-CT-2005–028,857, SHARELIFE: CIT4-CT-2006–028,812), FP7 (SHARE-PREP: GA N°211,909, SHARE-LEAP: GA N°227,822, SHARE M4: GA N°261,982, DASISH: GA N°283,646) and Horizon 2020 (SHARE-DEV3: GA N°676,536, SHARE-COHESION: GA N°870,628, SERISS: GA N°654,221, SSHOC: GA N°823,782, SHARE-COVID19: GA N°101,015,924) and by DG Employment, Social Affairs & Inclusion through VS 2015/0195, VS 2016/0135, VS 2018/0285, VS 2019/0332, and VS 2020/0313. Additional funding from the German Ministry of Education and Research, the Max Planck Society for the Advancement of Science, the U.S. National Institute on Aging (U01_AG09740-13S2, P01_AG005842, P01_AG08291, P30_AG12815, R21_AG025169, Y1-AG-4553–01, IAG_BSR06-11, OGHA_04-064, HHSN271201300071C, RAG052527A) and from various national funding sources is gratefully acknowledged (see www.share-project.org). Statistical model syntax is available from one of the authors, Małgorzata Kalbarczyk (mkalbarczyk@wne.uw.edu.pl) on reasonable request.

## Footnotes

1 Retirement was recorded if the respondent declared the year of retirement as 2012, 2013 or 2014.
